# Metabolite Profiling by UPLC-MS^E^, NMR, and Antioxidant Properties of Amazonian Fruits: Mamey Apple (Mammea Americana), Camapu (Physalis Angulata), and Uxi (Endopleura Uchi)

**DOI:** 10.3390/molecules25020342

**Published:** 2020-01-15

**Authors:** Larissa Gabrielly Barbosa Lima, Julia Montenegro, Joel Pimentel de Abreu, Millena Cristina Barros Santos, Talita Pimenta do Nascimento, Maiara da Silva Santos, Antônio Gilberto Ferreira, Luiz Claudio Cameron, Mariana Simões Larraz Ferreira, Anderson Junger Teodoro

**Affiliations:** 1 Laboratory of Functional Foods, Nutrition Biochemistry Core, Food and Nutrition Graduate Program, Federal University of the State of Rio de Janeiro, UNIRIO. Av. Pasteur, 296, Rio de Janeiro 22290-240, Brazil; larissagabrielly_lima@hotmail.com (L.G.B.L.); juliamontenegro95@gmail.com (J.M.); pimenabreu@gmail.com (J.P.d.A.); 2 Laboratory of Bioactives, Nutrition Biochemistry Core, Food and Nutrition Graduate Program, UNIRIO. Av. Pasteur, 296, Rio de Janeiro 22290-240, Brazil; barrosmillena@gmail.com (M.C.B.S.); talitapiment@gmail.com (T.P.d.N.); mariana.ferreira@unirio.br (M.S.L.F.); 3 Center of Innovation in Mass Spectrometry, Laboratory of Protein Biochemistry, UNIRIO. Av. Pasteur, 296, Rio de Janeiro 22290-240, Brazil; cameron@unirio.br; 4 Fluminense Federal Institute of Education, Science and Technology, IFF, Av. Dário Viêira Borges, 235-Lia Márcia, Bom Jesus do Itabapoana, Rio de Janeiro 28360-000, Brazil; maiarasantos@yahoo.com.br; 5 Laboratory of NMR, Department of Chemistry, Federal University of São Carlos, UFSCar. Washington Luiz, s/n, São Carlos 13565-905, SP, Brazil; giba_04@yahoo.com.br

**Keywords:** Amazonian fruits, antioxidant, phenolic compounds, UPLC-MS^E^, bioactive compounds

## Abstract

The metabolite profiling associated with the antioxidant potential of Amazonian fruits represents an important step to the bioactive compound′s characterization due to the large biodiversity in this region. The comprehensive bioactive compounds profile and antioxidant capacities of mamey apple (*Mammea americana*), camapu (*Physalis angulata*), and uxi (*Endopleura uchi*) was determined for the first time. Bioactive compounds were characterized by ultra-performance liquid chromatography coupled to high resolution mass spectrometry (UPLC-MS^E^) in aqueous and ethanolic extracts. Globally, a total of 293 metabolites were tentatively identified in mamey apple, campau, and uxi extracts. The main classes of compounds in the three species were terpenoids (61), phenolic acids (58), and flavonoids (53). Ethanolic extracts of fruits showed higher antioxidant activity and total ion abundance of bioactive compounds than aqueous. Uxi had the highest values of phenolic content (701.84 mg GAE/100 g), ABTS (1602.7 μmol Trolox g^−1^), and ORAC (15.04 μmol Trolox g^−1^). Mamey apple had the highest results for DPPH (1168.42 μmol TE g^−1^) and FRAP (1381.13 μmol FSE g^−1^). Nuclear magnetic resonance (NMR) spectroscopy results showed that sugars and lipids were the substances with the highest amounts in mamey apple and camapu. Data referring to chemical characteristics and antioxidant capacity of these fruits can contribute to their economic exploitation.

## 1. Introduction

The Amazonian region offers a wide variety of native fruits and most of them are typically obtained from nature or grown only for the local market supply in form of pulp or in natura. The economic exploitation is potentially of great importance for the region [1]. These native fruits have been studied as potential bioactive sources and many of them have shown high antioxidant capacity and elevated phenolic compounds content [2].

The antioxidant properties of Amazonian fruits have been the object of many researches, mainly due to the presence of natural antioxidants such as carotenoids and phenolic compounds. The group of antioxidant compounds found in fruits produced in Amazonian can protect the human body against toxic effects and preventing diseases such as chronic degenerative disorders, cardiovascular diseases, premature ageing, diabetes, and neurodegenerative diseases [3,4,5].

The Brazilian Amazonian region has a great biodiversity with approximately about 220 edible plant species producing fruit, representing 44% of native fruit diversity in Brazil. Some species such as mamey apple (*Mammea americana*), camapu (*Physalis angulata*), and uxi (*Endopleura uchi*) have widely appreciated flavors by Brazilian consumers, but still of moderate importance to the economy. They show potential for commercialization in both domestic and international markets [6].

Mamey apple (*Mammea americana*) is a fruit with a reddish yellow color, aromatic, and edible pulp, and is popularly known as abricó-do-pará, abricó, mammey apple or apricot from St. Domingo. This fruit has an important agroindustrial potential and this pulp can be used to produce different products such as syrup, juice, sherbet, jam, and pastes [7]. *Physalis angulata* L. is an herb indigenous of the *Solanaceae* family, and dispersed throughout tropical areas, including the Amazonian region [8]. This is popularly known as “camapu” and its juice is used as sedative, depurative, anti-rheumatic, and for the relief of earache, and is also used as a traditional medicine [9]. Uxi (*Endopleura uxi*) is an important fruit distributed in Pará and Amazonas, states of Northern Brazil. The only edible part of uxi presents a yellow-brownish color pulp with a rough-like texture, containing high content of fat (mainly oleic acid) and carotenoids, mostly trans-β-carotene, with an unique flavor [10].

Due to their peculiar biodiversity, knowledge of the species and functional property characterization of native Amazonian fruits such as mamey apple, camapu, and uxi present a major challenge to their appreciation, as many of these species are unexplored and their chemical properties remain unknown. 

Few studies in the literature were found to provide a comprehensive metabolomic analysis of the bioactive compounds combined to antioxidant capacity of these fruits from the Amazonian biome. However, some studies have shown the metabolomic composition of Amazonian native fruits. Paz et al. [11] studied *Clavija lancifolia Desf*. using liquid chromatography mass spectrometry (LC–MS/MS) and found some compounds of flavonoids for which kaempferol was the main compound. Souza et al. [12] identified the phenolic compounds cyanidin 3-*O*-rutinoside, chlorogenic acid, and rutin in *Oenocarpus distichus* fruits, using high-performance liquid chromatography (HPLC). It has been reported the presence of catechin, caffeic acid, rutin, orientin, quercetin, apigenin, luteolin, and kaempferol in *Mauritia flexuosa* L. f. (*Arecaceae*), another fruit from Amazonian biome [13].

Concerning the importance of the characterization of bioactive compounds from Amazonian fruits, the aim of this study was to assess the phytochemical profile and the total antioxidant capacity of mamey apple (*Mammea americana*), camapu (*Physalis angulata*), and uxi (*Endopleura uchi*).

## 2. Results and Discussion

### 2.1. Total Phenolic Compounds Content

**Figure 1:**
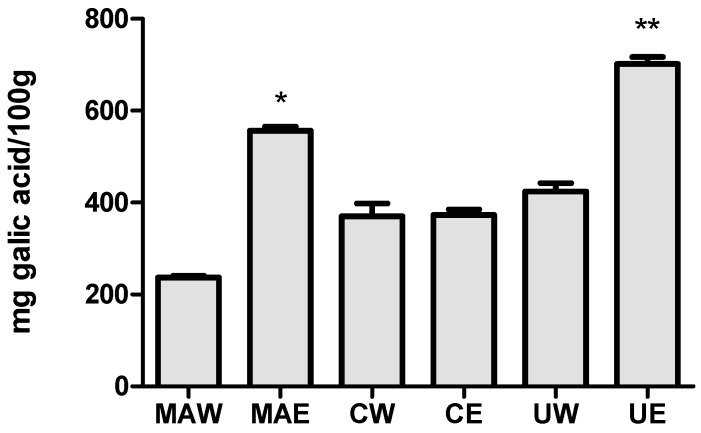
Total phenolic content in aqueous mamey apple (MAW), ethanolic mamey apple (MAE), aqueous camapu (CW), ethanolic camapu (CE), aqueous uxi (UW), and ethanolic uxi (UE) extracts. Results are expressed by mean ± SD (*n* = 3) and were compared by the one-way ANOVA test with post-test Tukey′s (* *p* < 0.01; ** *p* < 0.001).

Mamey apple (MA), camapu (C), and uxi (U) were submitted for analysis of total phenolic compounds from extractions submitted with different vehicles solvents (water (W) and ethanol (E)) to determine the most efficient extraction solvent of the mentioned compounds. The results showed that the ethanolic extracts (MAE and UE) of the different Amazonian fruits showed a greater quantity of phenolic compounds when compared to the aqueous extracts (MAW and UW) (Figure 1). The preparation and extraction from this wide range of samples depends mostly on the nature of the sample matrix and the chemical properties of the phenolics, including molecular structure, polarity, concentration, number of aromatic rings, and hydroxyl groups. Conventional solid-liquid using organic solvent extraction is the main method used to extract phenolics. The sample preparation, polarity of the solvent used, the technique employed and temperature are factors that can influence the extraction and contents of these compounds [14].

Uxi ethanolic extract (UE) showed the highest phenolic compound content (701.839 mg GAE/100 g), followed by mamey apple (MAE) (556.105 mg GAE/100 g), and camapu (CE) (237.39 mg GAE/100 g). The aqueous extracts of mamey apple (MAW) and uxi (UW) showed significant difference (*p* < 0.05) when compared to the respective ethanolic extracts. No significant differences (*p* > 0.05) were observed in the content of phenolic compounds between the aqueous and ethanolic extracts in the camapu sample.

Aqueous and ethanolic extract showed similar fruits yield extracts, being dependent on the moisture percentage of each fruit. Uxi yield was significantly higher (UE-28.82% and UW-29.42%), followed by mamey apple (MAE-14.04% and MAW-13.13%) and camapu (CE-10.50% and CW-11.10%). Considering these results, the levels of phenolic compounds per mass of fruits for the highest yield would be 202.27, 78.08, and 24.92 mg GAE/100 g for uxi, mamey apple, and camapu, respectively.

Péroumal et al. [15] studied the pulp of six mamey apple accessions and found values for total phenolic content between 90 and 143 mg GAE/100 g. It can be observed that the results obtained for uxi ethanolic extracts in this work are higher than some Amazonian fruits such as araça-boi (87.2 ± 3.0 mg GAE/100 g) and araça (129.1 ± 9.3 mg GAE/100 g), and lower than camu-camu (1797.2 ± 37.7 mg GAE/100 g) reported by Genovese et al. [16]. Comparing the results of this study with fruits from the Brazilian Cerrado biome, the Amazonian fruits present lower phenolic compounds in relation to sweet passion fruit (245.36 ± 3.70 mg GAE/100 g), soursop (281 ± 5.40 mg GAE/100 g), murici (334.37 ± 9.07 mg GAE/100 g), and marolo (739.37 ± 7.92 mg GAE/100 g) [17]. According to the classification proposed by Vasco et al. [18], MAE, uxi extracts were classified as fruits as having with medium phenolic content (100–500 mg GAE/100 g).

### 2.2. Phytochemical Profile by UPLC-MS^E^

For the first time, bioactive compounds of Amazonian fruits like mamey apple, camapu, and uxi were elucidated by UPLC-MS^E^ metabolomic approach. Globally, 293 compounds were tentatively identified from aqueous and ethanolic extracts of these fruits (Table S1) and relatively quantified taking account all extracts based on ion counting. Table 1 shows the number of identified compounds and their classification into eight chemical classes according to Phenol Explorer database [19]: phenolic acid, flavonoids, chalcones, coumarins, amino acid related compounds, fatty acid, and terpene related compounds. The main bioactive compounds in the three species were terpenoids (*n* = 61; 21%), phenolic acids (*n* = 58; 20%), and flavonoids (*n* = 53; 18%). Other metabolites were also identified such as other polyphenols (*n* = 50; 17%), including lignans, coumarins and tannins, and also other metabolites (*n* = 56; 19%) such as amino acid related, alkaloids and polyketides, showing the extraction and LC-MS methods were suitable to characterize different polarities compounds.

The identification of different phenolic compounds in this study makes it relevant, since the presence of these compounds has a range of bioactivities, as already reported in vitro and in vivo studies [20]. In a recent study, extracts of phenolic compounds from jatobá-do-cerrado can inhibit α-amylase and α-glycosidase after in vitro digestion and modulate the glucose metabolism [21].

Mamey apple was characterized by high number of bioactive compounds. Among the identified metabolites, 209 (71%) and 188 (64%) were found in aqueous and ethanolic extracts of mamey apple, respectively. Furthermore, 183 (62%) and 186 (63%) compounds were found in the aqueous extract ethanolic extracts of camapu, respectively. In uxi, 164 (56%) and 165 (56%) compounds were tentatively identified in the aqueous and ethanolic extracts, respectively. Notably, 50 (17%) compounds were found only in mamey apple (e.g., droserone, norlichexanthone, procyanidin C1, 3,4-leucopelargonidin II, and piquerol A). Piquerol A is a sesquiterpene with low stability in nature and has only previously been tested as an insecticide and as an inhibitor of metabolism in cell cultures [22]. About 43 (15%) were tentatively identified only in camapu (e.g., myristicin, 11-deoxocucurbitacin, synapic acid I, and pseudopurpurin). Medina et al. [23] reported the presence of sinapic acid in *Passiflora edulis Sims*. Furthermore, about 36 (12%) compounds were found exclusively in the uxi extract (e.g., 4-coumaroylchiquimate, ferulic acid I, kaempferol II, jacareubin, 6-deoxyjacareubin, vanylactic acid II, nigakilactone A, and jasmonic acid). Kaempferol is an important flavonol identified in other fruits like hybrid grapes by Rosso et al. [24].

These metabolites are antioxidant inhibitors of mutagenic and carcinogenic compounds and are considered neuroprotective agents in neurodegenerative disorders, such as Parkinson′s and Alzheimer′s diseases [25].

The acid phenolic profiling was the most abundant subclass of phenolic compounds in all extracts evaluated, as shown in the Table 1 and Table 2. Aqueous and ethanolic camapu extracts (CW and CE) showed about 24 and 22% of phenolic acids, respectively, followed by mamey apple aqueous (MAW) and ethanolic (MAE) extracts. In addition to phenolic acids, flavonoids showed high number of identification as well as elevated abundance relative.

Terpenes were between the most abundant bioactive classes in all extracts. About 46 and 43 terpenes, representing 22% of identifications, were found in the MAW and MAE extracts, respectively. In UE extracts, 39 (23%) terpenes were tentatively identified too. Terpenoids are important secondary metabolites of plants and are extremely chemically diversified, being estimated at more than 40,000 substances. Its use has been described as flavoring agents, in addition to providing benefits to human health through its antioxidant potential [26,27].

**Figure 2:**
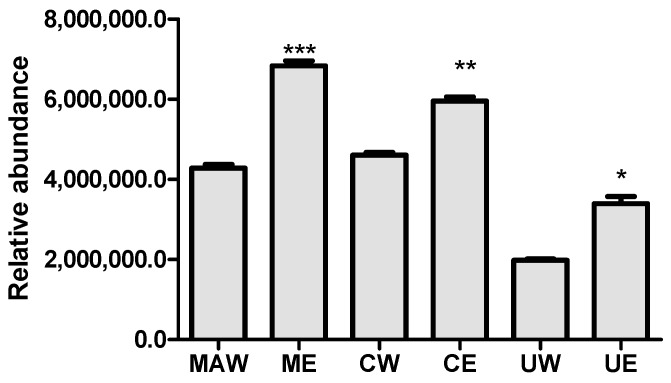
Relative abundance based on total ion counting of identified compounds from aqueous mamey apple (MAW), ethanolic mamey apple (MAE), aqueous camapu (CW), ethanolic camapu (CE), aqueous uxi (UW), and ethanolic uxi (UE) extracts. Results are expressed by mean ± SD (*n* = 3) and were compared by the one-way ANOVA test with post-test Tukey′s (* *p* < 0.05; ** *p* < 0.01; *** *p* < 0.001).

Although the number of identifications between the extracts of the same fruit was not significantly different, the ethanolic extracts showed a greater relative abundance of compounds in relation to the aqueous extracts in all fruits studied (Figure 2). This fact can be explained by the large variability of the structures of these bioactive compounds and the proportion of organic solvents with water is required for better extraction.

**Figure 3:**
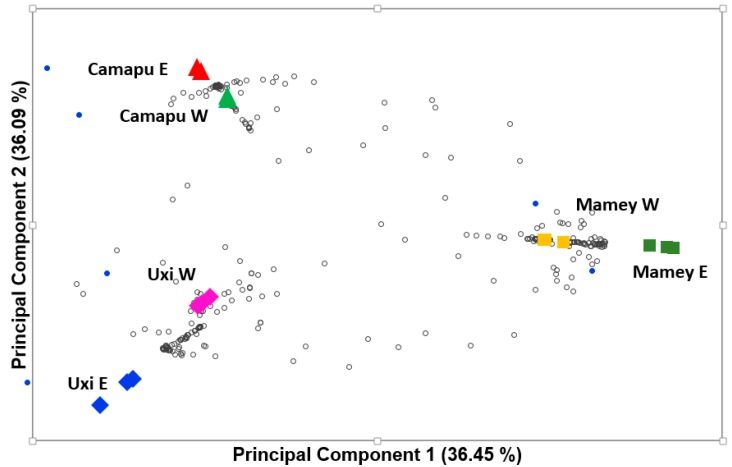
Principal component analysis (PCA) biplot (loadings and scores) of the bioactive compounds tentatively identified (loadings, empty circles) in the mamey (squares), camapu (triangles), and uxi (diamonds) fruits extracted with aqueous (W) and ethanolic (E) solvents.

**Figure 4:**
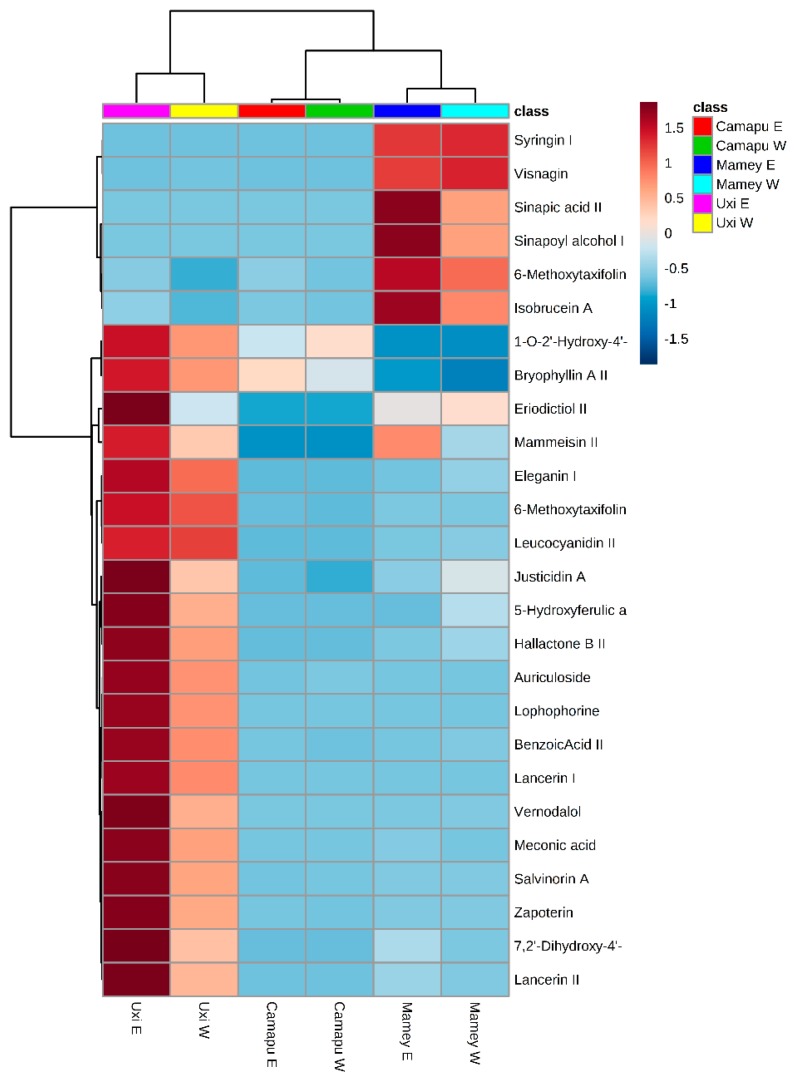
Hierarchical cluster analysis (HCA) and heatmap of the bioactive compounds in the mamey apple, camapu, and uxi fruits extracted with aqueous (W) and ethanolic (E) solvents, which showed maximum variance (eigenvectors) among extracts.

The principal component analysis (PCA) and hierarchical cluster analysis (HCA) were applied to explain possible difference among extracts (Figure 3 and Figure 4). First, the PCA biplot represented both loading (metabolites) and scores (extracts). Such parameters distinguished the profile of bioactive compounds among the fruits evaluated in all extracts. Principal components (PC1 and PC2) demonstrated that the fruits extracts have different composition of bioactive compounds, but the use of aqueous and ethanolic extractors did not favor the removal of distinct metabolite profile in the species. The two major principal components (PC1 and PC2) explained more than 72% of the variance pattern (Figure 3). Discriminatory metabolites, which showed maximum variance (eigenvalues) among extracts, were observed mainly in the PC2, including the following bioactive compounds bryophyllin A II, 1-*O*-2′-hydroxy-4′-methoxycinnamoyl-b-d-glucose I, mammeisin II, eriodictiol II, justicidin A, hallactone B II, 7,2′-dihydroxy-4′-methoxy-isoflavanol, eleganin I, 5-hydroxyferulic acid methyl ester II, lancerin II, 6-methoxytaxifolin II, zapoterin, salvinorin A, meconic acid, benzoic acid II, auriculoside, leucocyanidin II, lophophorine, lancerin I, vernodalol, visnagin, sinapic acid II, sinapyl alcohol II, 6-methoxytaxifolin I, isobrucein A, and syringin I. 

Then, HCA was applied to observe the similarity/dissimilarity among the abundance of the discriminatory polyphenols (Figure 4) in order to understand the profile of composition among extracts. The heatmap indicated that metabolites (20) found in uxi extracts showed higher abundance in comparison to other extracts, especially in the ethanol extracts. Mamey also showed a distinguished bioactive profile, with six compounds found exclusively. Among them, sinapoyl alcohol II is an exclusive metabolite with high abundance in the mamey apple extracts. One of the metabolites with highest abundance in these extracts was syringin. This compound was determined and reported from fresh jaboticaba methanolic extract by Wu et al. [28] and implicated as immunomodulator having an anti-allergic effect [29].

### 2.3. In Vitro Antioxidant Activity

Due to the multifunctional characteristics of the phenolic compounds found in Amazonian fruits, the effectiveness of measuring the antioxidant capacity of a pulp extract is better evaluated when using commonly accepted tests. The ethanolic and aqueous fruits extracts was determined by four different methods with different action mechanism (ABTS, DPPH, FRAP, and ORAC) (Table 3). According to Barros et al. [14], a single assay does not accurately account for all of the groups of antioxidant compounds, because of complexity of fruit matrices, and because of these methodologies can suffer interferences.

ABTS assay revealed values ranged between 263.67 ± 23.90 and 1602.7 ± 30 μmol Trolox g^−1^. The highest antioxidant capacity was presented in UE samples (1602.7 ± 30.16 μmol Trolox g^−1^). Freitas et al. [30] analyses fresh pulp of uxi and found 51.6 mg TE/100 g for ABTS. In general, the ethanolic extracts of fruits showed the higher antioxidant capacity for this methodology. However, the aqueous extract from camapu fruit was an exception with a value of 432.74 ± 16.17 μmol Trolox g^−1^ (Table 3). Schiassi et al. [31] studied the activity antioxidant of methanolic extracts of araça (10.92 ± 0.11 μmol Trolox g^−1^), buriti (6.03 ± 0.00 μmol Trolox g^−1^), cagaita (29.32 ± 0.69 μmol Trolox g^−1^), yellow mombin (5.55 ± 0.01 μmol Trolox g^−1^), and marolo (132.16 ± 1.40 μmol Trolox g^−1^) and found values lower than the present study.

Antioxidant capacity was also evaluated by DPPH^•^ radical scavenging method (Table 3) and expressed in aqueous and ethanolic extract concentration μmol Trolox g^−1^ sample. The highest activity was found in MAE (1168.42 μmol Trolox g^−1^), followed by CE (705.771 μmol Trolox g^−1^) and UE (509.68 μmol Trolox g^−1^). According to Huchin et al. [32], the DPPH method can analyze hydrophilic and lipophilic compounds, and ethanol is one of the most used solvents for bioactive compounds extraction due, because proposes some benefits, such as low toxicity, good extraction produce, it is safe for human consumption and allows the extracts to be used in the food industry [33].

Antioxidant activity by FRAP method followed the same pattern of the DPPH analysis, where higher values were found for the ethanolic extracts. It is probable that the antioxidant compounds of the samples detected by FRAP are the same with those evaluated by DPPH. MAE also presented the highest activity (1381.13 µmol ferrous sulphate g^−1^) These results was lower than obtained by Barros et al. [14] for aqueous extracts of achachairu (712.35 ± 6.61 μmol ferrous sulphate g^−1^), araça-boi (798.92 ± 1.52 μmol ferrous sulphate g^−1^), and bacaba (6567.45 ± 4.25 μmol ferrous sulphate g^−1^).

Regarding to ORAC assay, the results revealed that UE and UW obtained the highest value (15.04 ± 0.84 and 14.33 ± 1.36 μmol Trolox g^−1^), followed by CE and CW (11.15 ± 0.42 and 12.30 ± 1.15 μmol Trolox g^−1^). No significant differences (*p* > 0.05) were observed between the aqueous and ethanolic extracts of uxi and camapu sample. MAE (8.88 ± 0.52 μmol Trolox g^−1^) and MAW (5.17 ± 0.56 μmol Trolox g^−1^) showed the lowest values (*p* > 0.05) between extracts. Santos et al. [34] reported to Amazonian fruits such as bacaca (195.00 ± 10.00 μmol Trolox g^−1^), buriti (83.00 ± 6.00 μmol Trolox g^−1^), inajá (26.00 ± 2.00 μmol Trolox g^−1^), pupunha (94.00 ± 1.00 μmol Trolox g^−1^), and tucumã (64.00 ± 4.00 μmol Trolox g^−1^) results higher than camapu, mamey apple and uxi extracts.

### 2.4. Correlation between Total Phenolic Compounds and Antioxidant Capacity

The results of Pearson correlation coefficients (*r*) between total phenolic compounds and antioxidant capacity of aqueous and methanolic extracts suggest that the total phenolic compounds contributed to the in vitro antioxidant capacity of the extracts according to the method used. The aqueous extract showed a good association of total phenolic compounds with ABTS (*r* = 0.843) and ORAC (*r* = 0.752) for camapu fruit. ORAC assay has been largely applied to the assessment of free radical scavenging capacity of pure antioxidant compounds and antioxidant plant extracts [35]. The radical cation ABTS [2,29-]azinobis-(3-ethylbenzothiazoline-6-sulfonic acid) is one of the spectrophotometric methods used to measure water-soluble as well as lipid-soluble antioxidants, pure compounds, and food extracts. The pre-formed radical monocation of 2,29-azinobis-(3-ethylbenzothiazoline-6-sulfonic acid) (ABTS^•1^) is generated by oxidation of ABTS with potassium persulfate and is reduced in the presence of such hydrogen-donating antioxidants [36]. 1-*O*-2′-Hydroxy-4′-methoxycinnamoyl-b-d-glucose, a compound present in CW (Figure 4), this compound belongs to the sub class of organic compounds known as hydroxycinnamic acid glycosides. Hydroxycinnamic acids are natural antioxidants found in fruits, vegetables, and cereals [37].

Regarding to mamey-apple, the ethanolic extract presented positive correlation with total phenolic compounds in relation to DPPH assay (*r* = 0.910). The 2,2-diphenylpicrylhydrazyl (DPPH) assay is widely used in plant biochemistry to evaluate their properties for scavenging free radicals. The method is based on the spectrophotometric measurement of DPPH concentration change resulting from the reaction with an antioxidant [38].

According to Figure 4, the acids syrigin I and visnagin were components with high relative abundancy. Visnagin is an antioxidant furanocoumarin derivative and is a furanochromone that is furo [3,2-g]chromen-5-one which is substituted at positions 4 and 7 by methoxy and methyl groups, respectively. Syringic acid is a dimethoxybenzene that is 3,5-dimethyl ether derivative of gallic acid. It has a role as a plant metabolite, a member of benzoic acids, and derives from a gallic acid. Previous reports described that hydroxybenzoic acids itself and its derivatives showed antioxidant properties against different type of free radicals and can prevent or decrease overproduction of reactive species. The main structural feature responsible for the antioxidative and free radical scavenging activity in the case of phenolic derivatives is the phenolic hydroxyl group. Phenols are able to donate the hydrogen atom of the phenolic OH to the free radicals, thus stopping the propagation chain during the oxidation process. This effect is modulated by the ring substituents, so that electron-withdrawing groups increase the bond-dissociation enthalpy, due to the stabilization of the phenol by a polar structure that leaves a positive charge on the OH group [39,40,41].

In uxi ethanolic extract, some bioactive compounds were identified with great relative abundance, such as auriculoside, that is a flavan glycoside. Most natural flavans are lipid-soluble and prominent in fruit skin or peel and leaf surfaces, and are usually found at higher concentrations in immature fruits compared to mature fruits [42]. Lancerin a plant metabolite found in UE is a member of xanthones, a C-glycosyl compound and a polyphenol. Among the polyphenols, the xanthones derivatives comprise an important class of the oxygenated heterocycles with a diversity of substitution patterns that have been described for their antioxidant activity, show to act as metal chela-tors, free radical scavengers, as well as inhibitors of lipid peroxidation [43]. The presence of these compounds may have contributed to the strong correlation (*r* = 0.910) found between total phenolic compounds and ORAC assay.

These results demonstrate the importance of do different methods to available the antioxidant activity, above all in complex matrices. The positive correlation between compound phenolics and ORAC, can be explained due to this assay may be considered a more exact method, because it uses a biologically relevant radical source (peroxyl) and allows the measurement of total antioxidant capacity through the combination of the antioxidant capacity of hydrophilic and lipophilic fractions [44]. According to the methodology used, these results suggest that phenolic compounds may be one of the main factors responsible for the antioxidant capacity of mamey apple, camapu, and uxi.

### 2.5. NMR Profile

Table 4 described the identified compounds for each fruit, with the chemical shift and signal multiplicity, as well as coupling constant (*J*), and the quantification in mg/g of freeze-dried fruit. Camapu had the greatest number of different compounds, indicating that it has a large variety of nutrients and metabolites, even more than could be extracted and identified. Uxi had the least amount, probably due to the great quantity of lipophilic compounds that could not be extracted, so this result does not mean that there are few compounds in uxi.

Sugars were most abundant compounds in those fruits. Camapu showed the highest amount of sucrose, and mamey apple showed the highest amount of fructose and glucose, both α and β. In the uxi extracts no sugars were found. This result indicates that mamey apple and camapu are sweeter than uxi, and probably would be more accepted by consumers. The sugars present in mamey apple were sucrose (1.50 ± 0.10 mg/g), fructose (0.85 ±0.03 mg/g), α-glucose, and β-glucose (0.49 ± 0.02 and 0.40 ± 0.01 mg/g, respectively). The sugars found in camapu were sucrose (4.09 ± 0.46 mg/g), followed by fructose, α-glucose, and β-glucose (0.11 ± 0.00, 0.04 ± 0.00, and 0.04 ± 0.00 mg/g respectively).

Fruit and vegetable flavor depend upon taste (given by sweetness, sourness, acidity, and astringency) and aroma (concentrations of odor-active volatile compounds). Sweetness is determined by the concentrations of the predominant sugars, which are ranked relative to sucrose (fructose > sucrose > glucose). Sourness or acidity is determined by the predominant organic acids, which are ranked relative to citric acid (citric > malic > tartaric). Some amino acids, such as aspartic and glutamic, may also contribute to sourness. In general, consumer acceptance is related to soluble solids concentration (sugars, organic acids, soluble pectin, some phenolic compounds, and ascorbic acid) or the ratio of soluble solids to titratable acidity [45].

It has been shown that mamay apple has ethanol (0.01 ± 0.00 mg/g), which is possibly a fruit sugar fermentation product. It was also found choline (0.01 ± 0.00 mg/g) and organic acids, such as formic acid and shikimic acid (0.01 ± 0.00 and 0.11 ± 0.00 mg/g) in mamey apple. Shikimic acid (3,4,5-trihydroxy-1-cyclohexene-1-carboxylic acid) is natural acid from the plant metabolism and common in berries fruits. It is a precursor for the biosynthesis of primary metabolites such as aromatic amino acids and folic acid, and a great many other aromatic compounds. The benzene ring is formed through the shikimate pathway, and shikimic acid is an extremely essential compound in plants and microbes. Shikimic acid has been found to occur in many tissues of a variety of plants, with a sufficiently high percentage. Moreover, its content and accumulation in different tissues depends on the rate of metabolic processes taking place in them. Shikimic acid is utilized for industrial synthesis of the oseltamivir (antiviral), zeylenone (employed as a preparation for chemotherapy of cancer), and monopalmityloxy shikimic acid (anticoagulant activity). In addition, shikimic acid derivatives represent a great interest for agriculture because many of them are used as herbicides and antibacterial agents [46].

Choline is a natural amine that can be synthesized in human body, but this production usually is not enough to meet human needs in men and postmenopausal women. Some important functions of choline are it is a part of the neurotransmitter acetylcholine; it is a part of the predominant phospholipids in membranes; it forms betaine, which is an important osmolyte in the kidney glomerulus and helps with the reabsorption of water from the kidney tubule. Eggs and liver are the main sources of choline, but many other foods contain significant amounts of choline and esters of choline [47].

Ethanol (0.02 ± 0.00 mg/g) was also present in camapu, indicating fermentation. The organic acids found in camapu were lactic acid, acetic acid and formic acid (0.01 ± 0.00, 0.01 ± 0.00, and 0.01 ± 0.00). Besides those organic acids, it was found γ-amino butyric acid (GABA) in camapu (0.02 ± 0.00 mg/g). GABA is a four-carbon nonprotein amino acid and is widespread in bacteria, animals, and plants. GABA is naturally present in small quantities in plants, and is produced in response to anaerobic conditions, *γ*-radiation, low pH, low or high temperatures, and darkness, and by mechanical manipulation. Some functions of GABA in plants are: regulation of cytosolic pH, protection against oxidative stress, defense against insects, and the regulation of pollen tube growth and guidance [48]. In vertebrates is a neurotransmitter that is deficient in the brain of people with Alzheimer disease [49]. Camapu has been shown to be effective in delaying the development of this disease and in its treatment, by increasing the proliferation of neural stem cells in vivo [50] and also shown an anxiolytic effect [51] which the authors suppose is due to the presence of GABA agonists. Several amino acids such as aspartic acid, alanine, and valine (0.03 ± 0.00, 0.02 ± 0.00, and 0.01 ± 0.08 mg/g) and choline (0.01 ± 0.00 mg/g) were also present in camapu in sufficient quantities to be identified.

For uxi, the compound with in the highest amount was linoleic acid (1.79 ± 0.10 mg/g), which is the main fatty acid of ω-6 group. This result shows that there are fatty acids that are sufficient to appear in a spectrum of fingerprints, even though other lipids may not have been completely extracted from the sample. There is probably a low amount of sugar in uxi fruit because there are high amounts of lipids and usually sugar and lipids in the pulp of fruits are inversely proportional. However, it was found ethanol (0.15 ± 0.01 mg/g), indicating that some sugar that was present before it was fermented. It was also found enough amount of acetic acid (0.15 ± 0.00 mg/g) valine and alanine amino acids (0.05 ± 0.00 and 0.04 ± 0.00 mg/g, respectively).

## 3. Material and Methods

### 3.1. Standards and Chemicals

2,2-Diphenyl-1-picrylhydrazyl (DPPH^•^), 2,2′-azino-bis (3-ethylbenzothiazoline-6-sulfonic acid) diammonium salt (ABTS), gallic acid, quercetin, 2′,7′-dichlorofluorescin diacetate (DCFH-DA), L-(+)-ascorbic acid (AA). Phenolic standards (caffeic acid, (+)-catechin, ellagic acid, (−)-epicatechin, gallic acid, gentisic acid, 4-hydroxybenzoic acid, myricetin, pyrogallol, quercetin, and quercetin 3-*O*-glucoside) were purchased from Sigma-Aldrich (St. Louis, MO, USA). All the solvents used for the UPLC-MS^E^ analysis were of LC-MS purity grade and were also purchased from Sigma-Aldrich (St. Louis, MO, USA).

### 3.2. Samples

The fruits in natura of mamey apple (*Mammea americana*), camapu (*Physalis angulata*), and uxi (*Endopleura uchi*) were obtained from producing regions of Pará State, in the months of January and February 2017. They were transported in vacuum plastic containers to Federal University of the State of Rio de Janeiro (UNIRIO, RJ, Brazil) and stored at −18 °C. The part of the fruit used for extraction was carried out according to their consumption, for mamey apple only pulp, for camapu the whole fruit and for uxi pulp and bark.

### 3.3. Sample Preparation

Samples were extracted by means of 2 extraction solvents: (I) ethanol 70% and (II) water, from 5 g of sample and 100 mL of extraction solvent, followed by homogenization at room temperature (−20 °C) in a homogenizer TE-420 (Tecnal, São Paulo, Brazil) for 10 min in the absence of light. The samples were then centrifuged (Thermo Fisher Scientific, Bartlesville, CA, USA) (5000× *g*, 5 min, 20 °C) and filtered through analytical filter paper. The extraction solvents were evaporated using a rotary evaporator under vacuum (Savant, Thermo Scientific) and the extracts were then frozen at −80 °C in an ultra-freezer and lyophilized (Terroni^®^ LD 300, São Carlos, SP, Brazil) for 24 h. After this process, extracts were frozen at −20 °C until use in the experiments.

For UPLC-MS^E^ analysis, the extraction solvents were evaporated using a rotary evaporator under vacuum (Savant, Thermo Scientific, USA) at 40 °C and thereafter ressuspended in methanol/acetonitrile/water (2:5:93; *v*/*v*). Stock solutions (500 ppm) of 11 standards (caffeic acid, (+)-catechin, ellagic acid, (−)-epicatechin, gallic acid, gentisic acid, 4-hydroxybenzoic acid, myricetin, pyrogallol, quercetin and quercetin 3-*O*-glucoside from Sigma-Aldrich were prepared individually by dissolving accurately weighed amounts of standards in aqueous methanol an aliquot of each stock solution was mixed to achieve a mixed standard solution with a final concentration of 10 mg/L for each compound. Finally, extracts and standards were filtered through a 0.22 µm syringe filter and stored at −80 °C until UPLC-MS^E^ analysis.

### 3.4. Quantification of Total Phenolic Compounds

Total phenolic content was determined by the Folin–Ciocalteu method, which was adapted from Rocchetti et al. [52]. The extract and 2.5 mL Folin-Ciocalteu reagent solution 10% were combined and then mixed well using a Vortex. The mixture was allowed to react for 5 min then 2 mL of sodium carbonate 4% solution was added and mixed well the solution was incubated at room temperature (25 °C) in the dark for 2 h. The absorbance was measured at 750 nm using a spectrophotometer (Turner^®^ 340, Haverhill, MA, USA) and the results were expressed in mg gallic acid equivalents (GAE) per 100 g of extract using a gallic acid (2,5–50 µg/µL) standard curve [53].

### 3.5. Phytochemical Profile Characterization by UPLC-MS^E^

The UPLC-MS^E^ analyses were carried out according to Santos et al. (2018) [54] with slightly modifications. Two uL of extracts and standards were injected in triplicate into an Acquity UPLC (Waters, Milford, MA, USA) coupled to Xevo G2-S QTOF-MS/MS (Waters, Manchester, UK) system equipped with an electrospray ionization source (ESI) operating in negative ion mode. The column used was a UPLC HSS T3 C18 (100 mm × 2.1 mm, 1.8 µm) (Waters, Wexford, Ireland). The flow rate was 0.6 mL/min and the mobile phase gradient elution consisted of acidified water (5 mM ammonium formate and 0.3% formic acid, *v*/*v*) (pump A) and acetonitrile containing 0.3% formic acid (pump B), as follows: 97% A at 0 min, 50% A at 6.8 min, 15% A at 7.4–8.5 min, followed by an additional equilibration step 97% A at 9.1–12 min. Data were acquired using a multiplexed MS/MS acquisition in the extended mode with alternating low and high energy acquisition (MS^E^) 30–55 eV using ultra-high pure argon (Ar). The capillary and cone voltage were set at 3.0 kV and 30 V, respectively. The desolvation gas (N_2_) was set at 600  L/h at 450 °C, the cone gas was set at 50  L/h and the source temperature at 120 °C. Data acquisition was performed by using MassLynx 4.1 (Waters Corporation, Milford, MA, USA). To ensure accuracy and reproducibility, all acquisitions were performed by infusing lock mass calibration with leucine-enkephaline (Waters Corporation, Milford, MA, USA) (*m*/*z* 554.2615) at a concentration of 1.0 ng/L in acetonitrile: H_2_O (50:50, *v*/*v*) with 0.1% (*v*/*v*) formic acid at a flow rate of 10 μL/min.

The raw data of all replicates were processed with Progenesis QI v2.1 (Nonlinear Dynamics, Waters Corporation, UK) with the following conditions: centroid data, resolution full-width at half maximum (FWHM) of 50,000, deprotonated molecule [M − H]^−^. The identification of phenolic compounds was performed by searching for polyphenols with MetaScope, using a customized database of polyphenol compounds from PubChemID by using the following parameters: precursor and fragment mass error tolerance (5 and 10 mg/L, respectively), retention time limit 7.5 min. Target analysis was also applied for identification of the phenolic compounds by comparing the run parameters of phenolic standards such as the retention time, exact mass, mass error, and the MS-MS spectra, besides the other above mentioned parameters. The processed data were exported to XLSTAT software (Addinsoft, Paris, France), where the values of relative abundance obtained from each compound based on ion mass spectra counting were used to relative quantification and then to the multivariate statistical evaluation where Principal Components Analysis (PCA) biplot was elaborated. The Metaboanalyst 3.0 web server was used for analysis of multivariate data (HCA, hierarchical cluster analysis and heat map) where the eigenvectors of the correlation matrix was used to select the discriminatory bioactive compounds between the samples analyzed.

### 3.6. Analysis by Nuclear Magnetic Resonance Spectroscopy

For the NMR analyses, samples were prepared in triplicate. It was done by weighing 30.0 (±0.5) mg of freeze-dried samples (for each repetition), which was subjected during 10 min to shaking in a vortex with 0.8 mL of extractor solvent (D_2_O for abrico and camapu; methanol-d4 for uxi), with TMSP-d4 at concentration 0.02% (*m*/*v*) as internal reference and mass standard and, after, to ultrasonic bath during 10 min. Next, samples were centrifuged (13,000 RPM) at room temperature for more 10 min. A 0.6 mL supernatant aliquot was analyzed.

The ¹H-NMR spectra, as well as the 2D experiments, were conducted in the Bruker Avance III 9.4 Tesla (400 MHz for hydrogen frequency) spectrometer equipped with a PABBI probe (5 mm) and with an Automatic Tuning and Matching (ATMA) unit located in NMR Laboratory of the Chemistry Department at Federal University of São Carlos-UFSCar, Brazil. The TMSP-d4 was used to determine the 0 point in the chemical shift scale. 1H NMR spectra were registered at temperature of 300 K using 64 K data points and the standard Bruker pulse sequence (zgps).

All spectra were obtained through pre-saturation pulse sequence in order to suppress the residual water sign in the solvent. Spectra were acquired through 16 scans (NS), spectral window 15.0191 mg/L with 128,000 data points, receiver gain (RG) 80.6, relation delay of 50 s (D1) and acquisition time of 10.9 s (AQ). The exponential line broadening function value adopted for spectral apodization (LB) was 0.30 Hz.

The NMR data was analyzed in TopSpin Brucker Software. Quantification was performed by comparison of the compound′s signals area and the area of the TMSP-d4 signal to find out the compound′s mass since the TMSP-d4 mass is known. The equation used was: analyte mass = (analyte area × TMSP-d4 ¹H Nº × TMSP-d4 mass × analyte MM)/(TMSP-d4 area × analyte ¹H Nº × TMSP-d4 MM). The quantification is expressed in mg/g of freeze-dried fruit.

### 3.7. ABTS

For ABTS assay, the procedure followed the method of Thaipong et al. [55]. The stock solutions included 7.0 mM ABTS solution and 140 mM potassium persulfate solution. The working solution was then prepared by mixing the two stock solutions, 5 mL ABTS solution in 88 µL potassium persulfate solution, and allowing them to react for 16 h at room temperature in the dark. The solution was then diluted by mixing 1 mL ABTS solution with ethanol necessary to obtain an absorbance of 0.709–0.701 units at 734 nm using the spectrophotometer (Turner^®^ 340, Haverhill, MA, USA). The fruits extracts were allowed to react with 2.5 mL of the ABTS solution for 6 min in a dark condition. The standard curve was linear between 0 and 2000 µM Trolox equivalents. Results are expressed in µM Trolox equivalents g^−1^ of extract.

### 3.8. DPPH

The antioxidant activity of all fruits extracts in relation to the DPPH (2,2-diphenyl-1-picrilidrazil) radical was quantified by using a protocol described by Brand-Williams and Berset [56], using wave 515 nm in spectrophotometer (Turner^®^ 340, Haverhill, MA, USA). The working solution was obtained by dissolving 2.4 mg DPPH reagent with 100 mL methanol and stored in the dark until needed. The extracts were allowed to react with 2.5 mL of the DPPH solution for 30 min in the dark. The standard curve was linear between 0 µM and 2000 µM Trolox. Results are expressed in µmol Trolox g^−1^ of extract.

### 3.9. FRAP

The FRAP assay was done according to Thaipong et al. [55]. The stock solutions included 0.3 M acetate buffer, pH 3.6, 10 mM TPTZ (2,4,6-tripyridyl-s-triazine) solution, and 20 mM ferric chloride solution. The fresh working solution was prepared by mixing 25 mL acetate buffer, 2.5 mL TPTZ solution, and 2.5 mL ferric chloride solution. Fruits extracts were allowed to react with 2.7 mL of the FRAP solution for 30 min in the dark condition and warmed at 37 °C. Readings of the colored product were then taken at 595 nm in spectrophotometer (Turner^®^ 340, Haverhill, MA, USA). The standard curve was linear between 500 µM and 2000 µM ferrous sulphate. Results are expressed in µmol ferrous sulphate g^−1^ of extract.

### 3.10. ORAC

The ORAC procedure used an automated plate reader (SpectraMax i3x, Molecular Devices, USA) with 96-well plates [54,55,56,57]. Analyses were conducted in phosphate buffer pH 7.4 at 37 °C. Peroxyl radical was generated using 2, 2′-azobis (2-amidino-propane) dihydrochloride which was prepared fresh for each run. Fluorescein was used as the substrate. Fluorescence conditions were as follows: excitation at 485 nm and emission at 520 nm. The standard curve was linear between 1 µM and 90 µM Trolox. Results are expressed as µM TE g^−1^ of extract.

### 3.11. Statistical

Statistical comparisons were carried out by ANOVA and post hoc Tukey′s test using Graph Pad Prism 5.0 and the differences were considered significant when *p* < 0.05 in total phenolic content and relative abundance of identified compounds. The correlation coefficients between different results of evaluation antioxidant capacity of the samples, ABTS, DPPH, FRAP, ORAC and total phenolic compounds (PC) were obtained. Principal component analysis (PCA) and heatmap was used to interpret data, using Stat graphics software.

## 4. Conclusions

This work highlights the significantly concentration of phenolic compounds and antioxidant properties of Amazonian fruits. A total of 293 phenolic compounds were tentatively identified in mamey apple, camapu, and uxi by comparison with standards and by fragmentation patterns. Mamey apple extracts presented major number of phenolic compounds, being the phenolic acids and terpenoids the classes more identified. ABTS, DPPH, FRAP, and ORAC assays revealed that the ethanol extract presents a higher antioxidant activity and aqueous extract presents a high correlation with phenolic compounds content in Amazonian fruits. The findings of this study highlight the potential of mamey apple, camapu, and uxi as a valuable source of natural antioxidants.

## Figures and Tables

**Table 1:** Number of bioactive compounds distributed by classes and other compounds identified in aqueous (W) and ethanolic (E) extracts of mamey apple, camapu, and uxi.

Compounds (%)	Mamey Apple		Camapu		Uxi	
	W	E	W	E	W	E
**Phenolic Acid**	43 (20.57%)	37 (19.68%)	44 (24.04%)	40 (21.51%)	38 (23.17%)	35 (21.21%)
**Flavonoids**	36 (17.22%)	30 (15.96%)	33 (18.03%)	29 (15.59%)	24 (14.63%)	27 (16.36%)
**Chalcones**	2 (0.96%)	2 (1.06%)	2 (1.09%)	2 (1.08%)	3 (1.83%)	3 (1.82%)
**Coumarins**	16 (7.66%)	15 (7.98%)	14 (7.65%)	14 (7.53%)	8 (4.88)	9 (5.45%)
**Others phenolic compounds**	27 (12.92%)	25 (13.30%)	23 (12.57%)	30 (16.13%)	20 (12.20%)	26 (15.76%)
**Amino acid related compounds**	17 (8.13%)	14 (7.45%)	17 (9.29%)	20 (10.75%)	18 (10.98%)	15 (9.09%)
**Fatty acids related compounds**	22 (10.53%)	22 (11.70%)	11 (6.01%)	12 (6.45%)	14 (8.54%)	15 (9.09%)
**Terpenoids**	46 (22.01%)	43 (22.87%)	39 (21.31%)	39 (20.97%)	39 (23.78%)	35 (21.21%)
**Total of compounds**	209 (100%)	188 (100%)	183 (100%)	186 (100%)	164 (100%)	165 (100%)

**Table 2:** Flavonoids tentatively identified in mamey apple, camapu, and uxi extracts.

Possible Identifications	CAS	*m*/*z* Exp	RT (min)	Fragment *m*/*z*	Error (ppm)	Mamey		Camapu		Uxi	
						W	E	W	E	W	E
**Genistin**	529-59-9	431.0978	4.55	**133.0294 (10.20)**	−1.23	−	−	−	−	X	X
**Eriodictiol I**	552-58-9	287.0552	3.05	81.0338 (5.46); 93.0339 (78.57); 119.0496 (100); 155.0342 (10.19); 163.0395 (60.20)	−2.86	X	X	−	−	−	−
**Hesperidin**	520-26-3	609.1875	0.43	79.0188 (0.66); 369.0671 (50.27); 488.1618 (10.04)	8.37	X	X	X	X	X	X
**Narirutin**	14259-46-2	579.1775	0.43	72.9924 (78.62); 79.0188 (0.58); 117.0187 (26.50); 135.0294 (87.65); 357.1033 (3.92); 369.0671 (44.06); 535.1514 (21.08)	9.66	X	X	X	X	X	X
**Pomiferin I**	572-03-2	419.1505	2.62	nd	1.21	X	X	X	X	X	X
**Ononin**	486-62-4	429.1170	2.02	nd	−4.89	X	X	X	X		
**Mammeisin**	18483-64-2	405.1677	4.01	109.0653 (0.46); 154.0615 (0.72)	−7.30	X	X				
**Quercitrin**	522-12-3	447.0923	3.48	151.0030 (2.20); 285.0393 (5.36)	−2.09	X	X	X			
**Quercetin 3-galactoside**	482-36-0	463.0875	3.47	151.0030 (2.97); 255.0291 (5.58); 271.0241 (16.99); 285.0393 (7.25); 300.0266 (31.67)	−1.44	X	X	X	X	X	X
**Kaempferol I**	520-18-3	285.0396	4.90	nd	−2.70	X	X				X
**Dihydroquercetin**	480-18-2	303.0505	1.42	147.0120 (100)	−1.65	X	X				
**Luteoforol**	24897-98-1	289.0733	0.56	109.0289 (12.30)	5.54	X	X	X	X	X	X

**Table 3:** Antioxidant activity of the mamey apple, camapu, and uxi samples by ABTS, DPPH, FRAP, and ORAC assays.

Assay	Mamey Apple		Camapu		Uxi	
	Aqueous	Ethanolic	Aqueous	Ethanolic	Aqueous	Ethanolic
**ABTS (μmol Trolox g^−1^)**	263.67 ± 23.90 ^a^	937.66 ± 218.49 ^b^	432.74 ± 16.17 ^c^	419.43 ± 18.55 ^c^	271.86 ± 22.14 ^a^	1602.7 ± 30.16 ^d^
**DPPH (μmol Trolox g^−1)^**	336.60 ± 3.05 ^a^	1168.42 ± 218.56 ^b^	386.24 ± 116.99 ^c^	705.77 ± 100.74 ^d^	46.95 ± 17.17 ^e^	509.27 ± 26.95 ^f^
**FRAP (µmol ferrous sulphate g^−1^)**	564.18 ± 18.90 ^a^	1381.13 ± 189.95 ^b^	970.60 ± 28.92 ^c^	1183.98 ^c^ ± 46.62 ^b^	376.66 ± 1.81 ^d^	448.68 ± 41.97 ^e^
**ORAC (µmol Trolox g^−1^)**	5.17 ± 0.56 ^a^	8.88 ± 0.52 ^b^	12.30 ± 1.15 ^c^	11.15 ± 0.42 ^c^	14.33 ± 1.36 ^d^	15.04 ± 0.84 ^d^

Results are expressed as mean ± standard deviation. Different letters on the same line show significant difference. Results were compared by the One-way ANOVA test with Tukey post-test (*p* < 0.05).

**Table 4:** NMR fingerprinting identification and quantification of mamey apple, camapu, and uxi.

Fruit	Compound	*δ* ¹H (ppm)	Multiplicity (J)	Mass (mg/g)
**Mamey Apple**	Formic acid	8.44	s	0.01 ± 0.00
	Shikimic acid	6.48	m	0.11 ± 0.01
	Sucrose	5.42	d (3.88)	1.51 ± 0.11
	α-glucose	5.22	d (3.76)	0.49 ± 0.02
	β-glucose	4.64	d (7.94)	0.40 ± 0.02
	Fructose	4.10	d (3.42)	0.85 ± 0.04
	Choline	3.19	s	0.01 ± 0.00
	Ethanol	1.18	t (7.09)	0.01 ± 0.00
**Camapu**	Formic acid	8.44	s	0.01 ± 0.00
	Sucrose	5.42	d (3.91)	4.09 ± 0.46
	α-glucose	5.23	d (3.73)	0.04 ± 0.01
	β-glucose	4.65	d (7.92)	0.04 ± 0.01
	Fructose	4.11	d (3.42)	0.12 ± 0.01
	Choline	3.20	s	0.01 ± 0.00
	Aspartic acid	2.82	dd (17.4; 3.78)	0.04 ± 0.01
	Acetic acid	1.94	S	0.01 ± 0.00
	GABA	1.89	quin (7.44)	0.02 ± 0.01
	Alanine	1.47	d (7.2)	0.02 ± 0.01
	Lactic acid	1.33	d (6.68)	0.01 ± 0.00
	Ethanol	1.18	t (7.09)	0.02 ± 0.001
	Valine	1.04	d (7.07)	0.01 ± 0.00
**Uxi**	Linoleic acid	5.32	M	1.79 ± 0.11
	Acetic acid	1.94	S	0.16 ± 0.01
	Alanine	1.45	d (7.20)	0.04 ± 0.01
	Ethanol	1.16	t (7.10)	0.15 ± 0.02
	Valine	1.05	d (7.05)	0.05 ± 0.001

## Data Availability

All the processed data are presented in the article. Information on raw data and materials are available from the corresponding authors upon request.

## Supplementary Materials

The following are available online. Table S1: Bioactive compounds detected and characterized in mamey apple, camapu and uxi fruits extracted with aqueous (W) and ethanolic (E) solvents.
